# Observations on Scarlet Fever

**Published:** 1833

**Authors:** Silas Reed

**Affiliations:** Cincinnati


### WESTERN JOURNAL

OF THE

### MEDICAL AND PHYSICAL SCIENCES.

APRIL, MAY AND JUNE, 1833.

### Essays and Cases.

ART. I.—*Observations on Scarlet Fever.* By Dr. SILAS REED. *Read before the Cincinnati Medical Society.*

MR. PRESIDENT:

I do not appear before the Society this evening, in the hope of throwing any new light upon the *nature* and *treatment* of Scarlet Fever. Too many men of talent and experience have already labored to illustrate these points, to afford me an opportunity of advancing any thing original upon them.

I propose, therefore, to confine my remarks principally to the history of the disease, as it appeared in the Western Reserve, Ohio, in the year 1831; and, by describing and enforcing the plan of treatment found most successful in its cure, I hope to contribute something to the support of the principles laid down for its treatment, by Dr. Armstrong, whom I regard as one of the most able medical philosophers of modern times.

EPIDEMIC CONSTITUTION.

Before proceeding to the consideration of the disease, it may be proper to say a few words upon the subject of *Epidemic Constitutions*, or the prevailing diathesis, which, unfortunately, is too little regarded by many at the present day. I know physicians who cannot believe, that diseases of the same name, may vary, essentially, in their character, in different years, and consequently require very different, and even opposite, modes of treatment.

Were this subject more generally studied, and more frequently permitted to govern our practice, we should be better able to explain *seeming* contradictions in our therapeutic observations.

Without an acquaintance with this important fact, young practitioners, who have never had an opportunity of witnessing epidemics of a lower grade of action, will frequently become exceedingly embarrassed in the treatment of those several conditions of the system, which usually manifest themselves during the prevalence of a highly asthenic diathesis.

During the reign of all epidemics, whether they assume the sthenic or asthenic diathesis, special regard should be paid to the prevailing epidemic constitution, which possesses such a commanding influence over all diseases. Perhaps no physician ever paid more attention to this subject, than the great Sydenham. His observations embraced almost every circumstance of any importance, connected with epidemic diseases; and to him, I believe, we are more indebted for an improved practice in tonic as well as atonic diseases, than to any other man. What constitutes the epidemic constitution, which is capable of producing at one period a tonic, and at another an atonic diathesis of the system, is difficult to say. The peculiarities of the general state of the atmosphere, which contribute to the production of these effects, cannot be satisfactorily explained, from their being altogether uncognizable by our senses, yet we should by no means neglect to watch their effects, especially when *local causes* are present, which always adds complexity and inveteracy to the disease arising from their union. To illustrate this, I would refer to the choleric diathesis, which has been spreading its desolating effects over our country during the nine months past. To any one who has noticed the rise and progress of the cholera through Canada and the United States, and the epidemic disposition to the disease which prevailed among us during the early part of last summer, it will not be doubtful, I think, that an insensible distemperature of the atmosphere prevailed, which only required some unknown *local cause*, (which seemed to arise more particularly along the larger water courses,) to light up the disease. The disposition to cholera has, in a measure, subsided: but its atonic influence has by no means, passed from among us. Scarlet fever is now prevailing. It will continue to prevail for some time to come—though it will be variable in its progress—at one time almost disappearing, at another breaking forth with renewed violence—until summer shall come upon us, with all its miasmata and other local causes, and perhaps add greatly to its malignity.

This may seem to savour too much of the spirit of prophecy; but when it is considered that Scarlet Fever generally lingers about certain localities for a considerable length of time; that its progress is very slow; and that the summer and autumnal months increase very much its prevalence, and add exceedingly to its violence and complication—I think I shall not be thought to merit such an imputation.

We have the high authority of the learned Noah Webster and several others, in favor of the doctrine of meteorations co-operating with local causes, in the production of various epidemics. That there is an occasional prevalence of a peculiar condition of the insensible properties of the atmosphere, favoring the prevalence of, or giving rise to, particular epidemics, is now, I believe, pretty generally acknowledged.

This change in the insensible properties of the atmosphere, has been termed by Dr. J. M. Smith, "epidemic or insensible meteoration," as distinct from *sensible* meteoration, or changes from heat to cold, dryness to moisture, &c. &c.

There can be no doubt but an epidemic constitution of the atmosphere, whatever it may be, immediately precedes or accompanies all epidemics—producing in the system a certain exaltation or subjugation of the vital powers, which favors, as they arise, the morbid influence of certain local causes, and a disease of a high or low grade of action, according as the diathesis of the system is either *tonic* or *atonic*, becomes established. The nature of this operating agent in the atmosphere, says Dr. Stephen Brown, forms a subject of curious, interesting, and philosophical investigation to every inquiring mind.

TOPOGRAPHY OF THE WESTERN RESERVE.

Before proceeding to remark upon the outbreaking of the disease, I must beg your indulgence a few moments, while I take a cursory glance at the topography of the Western Reserve. To many it has appeared surprising that this salubrious section of country should have been so frequently visited by epidemics. But the artificial production of local causes by what are denominated improvements, may in some measure, account for the fact.

It embraces the northeast section of the great ridge, which divides the waters of Lake Erie from those of the Ohio. The greatest height of this ridge lies 35 miles from the Lake, running parallel with it, and rising upwards of 400 feet above its waters. But the descent, both towards the Lake and River, is so gradual as to give the country the appearance of being almost as level as a plain.

It is, however, nearly destitute of marshes, and to a casual observer, or one little acquainted with the history and topography of this interesting section of our State, the frequency and malignity of its epidemics, could in no way be accounted for. But it must be recollected that the Big-Beaver and Muskingum rivers, take their principal,—and the Cuyahoga and Grand rivers, their entire rise, from the numerous small lakes which are found lying upon the summit of this ridge.

The course of three of these streams being short, and the fall consequently great, affording numerous mill seats, it might be expected that artificial reservoirs, occasioned by milldams, would become very much multiplied. This has been the case; and ever since the close of war, in 1815, when these water powers began to be improved, the epidemics on the Reserve have increased in frequency, and mortality. About the several heads of the Cuyahoga, where the Scarlatina prevailed when it fell under my observation—the number of natural and artificial reservoirs of water has, for many years, been unusually great—and, until within two or three years, the mill ponds were permitted to be kept up during the summer and autumnal months. It will not appear strange then, that miasmata should be generated liberally, that fevers should prevail every year—and that they should partake much of the congestive character. For many years marsh miasma was so abundant as to give rise to a constant *paludal diathesis* which appeared to act as a continual predisposing cause to various epidemics—and epidemics or insensible meteoration being only required to call them forth.

I am inclined to the belief, that local causes more frequently give the *character* to epidemics—than general causes. But with regard to the local causes which I have mentioned—few *now* exist. The people in the abundance of their sad experience, learned wisdom—and these artificial reservoirs, either from popular inclination or judicial decisions, are now destroyed during the summer months.

During the winter of 1813—14, the pnuemonia typhoides prevailed to an alarming extent on the Western Reserve, as in other parts of the United States. It was a highly congestive and fatal disease—and the first, I believe which denoted a change from the inflammatory to a typhoid diathesis, in that section of country. This diathesis continued to prevail more or less until 1830—but the grade of action frequently varied during this long period.

The great length of time which this diathesis continued, resulted, principally, I am inclined to think, from the universal, and almost constant prevalence of local causes—as marsh miasmata, &c.

The typhoid dysentery of 1824, 26, and 28, which destroyed so many hundred children—and the malignant fevers of 1825, 27, 28, and 30, which were so fatal, and occasioned so much speculation and controversy between the advocates of stimulants in

"*Typhus Syncopalis*," and their opponents—undoubtedly derived their malignity from a sigular co-operation of *general* with *local* causes—occasioning that remarkable prostration of vital action which is observable at the outset of all highly asthenic or congestive diseases.

Who does not recollect the winter and summer of 1828? To the people of the Reserve, it was a year that will long be remembered. The mortality which occurred in Middleburg village—during the months of August and September was truly appalling. It was thought to be typhus syncopalis, and treated accordingly—little regard being paid to the pathological conditions of the several stages and varieties. Its mortality greatly exceeded that of the cholera in this city—being six out of every hundred. I mention these circumstances in order to show the character of the diathesis, which so closely preceded the commencement of scarlet fever, in that locality. The diathesis, however, seems to have become much milder since that year.

### Outbreaking of the disease.

It was in December, 1830, that Scarlatina first appeared in Portage county, and the first case which occurred, happened in my own practice—but it was so very mild, as to induce me to think it a common urticaria. The next did not occur in my practice. The patient was a girl of 8 or 10 years of age, and was taken with the usual symptoms of oppression—which continued three or four days—when, by the use of mild purges, and by the natural energy of the system, reaction was induced, and an inflammation of the tonsils came on, together with a faint and partial eruption on the surface. Her physician having never seen the disease, feared to proceed boldly with his treatment lest debility might be induced. The excitement proceded and inflammation of the brain ensued; when a small bleeding was used—but *too small* to be of the least advantage. Blisters were then applied, but the inflammation progressed, and the girl died with more strength to the last, than she possessed when taken sick.

Had a more vigorous treatment been employed at the commencement—or had the lancet been used with greater freedom after local inflammation occurred, I am inclined to believe that the patient might have been saved. On the morning after the death of the daughter, her brother, a boy 5 or 6 years of age, was taken with restlessness, nausea, languor, and debility. The father fearing a similar, but more violent attack than occurred in the girl, determined to commence the treatment of the case himself, and thereby nip the disease in the bud. Accordingly, during the forming or highly congestive stage of the disorder, he administered a strong decoction of the *eupatorium perfoliatum*, without premising a foot bath, and warm stimulating drinks. Severe emesis and catharsis ensued, which rapidly exhausted the powers of life,—coma and collapse supervened, and the little patient slept in death within ten hours after the attack. Had the proper means of producing reaction, been used at the commencement, I believe it might have been raised, after which the disease would have run through a course of considerable excitement, and nothing been heard about malignity, debility and typhus.

It is all-important that we adapt our remedies, to the several stages and varieties. Scarlatina, as it appeared on the Western Reserve, was generally a disease of a highly adynamic character; and in those cases in which reaction was developed, it rarely continued many hours without spreading its violence upon some feeble organ. It was necessary to use stimulation and counteraction one hour—and the next employ depletion.

On the day following the death of the boy, another sister, three years of age, sickened. The parents by this time were panic struck, and began to think their children had taken poison. As the physician called in despaired of her recovery, for no other reason than that the other two had died, several other physicians were summoned, and after using the warm bath, sinapisms, warm drinks, and 20 grains of calomel to evacuate the bowels, the eruption appeared, and in three days she recovered, having had the simple variety, with but little affection of the throat.

I have been thus particular in the mention of the preceding cases, in order to show how the disease commenced—an inflammatory, congestive, and simple variety occurring in the same family.

Most of the cases which occurred during the winter and following spring, assumed the simple form—and were very manageable. Towards summer it increased in violence—but the mortality was slight. As autumn approached, the disease seemed to ingraft itself upon the prevailing remittent diathesis, and the complexity and inveteracy which followed, rendered the treatment exceedingly troublesome, and very much increased its mortality.

### Varieties and Stages.

It appears to me that we should adopt Dr. Armstrong's division of the disease into the simple, inflammatory, and congestive varieties, instead of simple, anginose, and malignant. The anginose affection is more or less present in all the varieties; and the malignant form is as frequently the consequence of unsubdued reaction, as of original reduction of vital energy. For practical purposes, it is important that we should attach such *names* to diseases, as are best calculated to convey some idea of their nature—hence the propriety of Armstrong's division. The reaction of the second variety can hardly be said to be *inflammatory*, it is of an adynamic character, and little else than an exalted grade of simple excitement. This reaction exists, however, for so short a time before the development of *local* inflammation, that a better term could not well be substituted. It would be quite unnecessary to describe the symptoms of the several varieties—they answered to the description given by Armstrong. Stiffness of the neck, and swollen and inflamed tonsils, were the first symptoms that indicated the approach of the disease; many, however, had the anginose affection without any attending eruption, and, among adults it was not uncommon to find the eruption without any affection of the throat. The most blooming and general efflorescence occurred in the simple variety—or where local inflammation was not present—

and was always considered a favorable sign. It seldom appeared in the congestive variety, unless the circulation became equalized, and reaction was established; and even then, it frequently assumed a purplish hue, denoting a remarkable atony and relaxation of the capillaries.

A tumefaction of the hands and feet, usually arose with the disease, though dropsical swellings were rarely the consequence. It was not unusual to meet with rheumatic affections, as the result of unsubdued excitement or slight exposure—but they were easily checked by the use of the vol. tinct. Guaiacum. The stage of oppression was short and slight in the simple variety—but long and severe in the congestive.

It happened not unfrequently, that the children of a whole family would be attacked with it, almost simultaneously—while those of another, would sicken at intervals of two or three weeks. A part of the children of some families escaped entirely.

The degree of subsequent reaction could be measured pretty accurately, by the violence and duration of the stage of oppression. The longer and more severe this stage, without inducing congestion, the more violent and malignant would be the following reaction. The stage of oppression tends much to enfeeble the internal organs, and prostrate the vitality; and the diathesis is such, that if reaction supervene, it will most generally eventuate in local inflammation. Hence the great necessity of watching this disposition at the very commencement of reaction, especially during that season of the year when the remittent diathesis prevails, or we shallfind it very difficult—many times even impossible, to effect a cure.

The organs most likely to suffer from inflammation and congestion during the course of the disease, are the brain, the liver, and the tonsils.

Excitement, followed by local inflammation, was the most common form of the disease during the summer and autumnal months. I do not recollect meeting with any well marked cases of the true congestive variety, as described by Dr. Armstrong, except as the result of bad management. A few cases occurred, which wore the aspect of his *irregular* variety, when the stage of oppression was so very severe and protracted, as to be followed only by a slight and feeble excitement. The most strongly marked case of this kind which was recovered from, happened in a boy of nine years of age. The attack was so severe as to induce the parents to think him dying. When I first saw him, he was speechless, stupid, and cold; appearing much like one in a state of syncope—the face was pale, the lips livid, and the pulse almost imperceptible to the touch. He was immediately placed in a warm bath, ammonia, camphor, capsicum, and brandy toddy, were given; mustard plasters and bottles of hot water were afterwards applied, when a slight reaction arose, and continued for several days. Great torpor of the liver and disposition to cerebral congestion were present, but they finally disappeared under a liberal use of calomel and blisters. This boy now resides in Covington, Kentucky. He was taken several weeks after two of his younger brothers, who had the simple variety.

### Pathology.

The pathology of this disease is similar to that of all adynamic fevers, which present the several varieties of simple excitement, inflammation, and congestion. These varieties and grades of severity, depend more on the degree of overthrow in the capillary function, than on the presence or absence of venous congestion. Though Armstrong's treatise on this disease is the best I have had an opportunity of reading, yet he appears too much inclined to confine his pathological views to the various derangements of the large vessels. I am disposed to think, that we should view their derangements, whether inflammatory or congestive, as the *effects* of disordered capillary action—rather than the *cause*. Had he taken this view of the pathology, he probably would have been less inclined to bloodletting in the congestive variety and stage of this disease. The various kinds of counter-irritants, seem to be of more service in restoring a healthy condition of the capillaries in congestion, than bloodletting; except the congestion follow a general excitement.

The difference between the inflammatory and congestive forms of disease, seems to me, to depend, not only on a morbid state of the capillaries of a *greater number* of organs in the latter variety, but upon a greater degree of atony induced in these small vessels. When a sufficient tonicity of the capillaries prevails, excitement will be the result; but when they labor under a high degree of atony, congestion of the large vessels, collapse, and death will ensue; unless efficient counteraction, by external and internal stimulation succeed in restoring their tone, and equalizing the circulation.

We are too prone to overlook the nervous and capillary derangement in disease. Their functions are the first to become disordered—and the first which demand restoration.

In the inflammatory variety, there is less waste of the excitability—the small and large vessels possess a tolerable share of tonicity—and excitement is the result. In the congestive form, the sensorial power is deficient, the capillaries appear paralyzed; the action of the heart is enfeebled; the secretions become altered or suppressed; the healthy balance of the circulation is destroyed; and coma and collapse are the consequence.

### Treatment.

From the foregoing remarks, it will at once be seen that the remediate management of the disease must be greatly modified, by the different varieties which it is apt to assume. The nature of the cause, the sthenic or asthenic diathesis of the system, and the varieties and stages of the disease, are to be taken into consideration. The complaint generally discloses great want of resistance in the system, yet, if the stage of excitement be not carefully watched, and kept sufficiently subdued, we need place little reliance upon the success of our subsequent treatment.

Before I had an opportunity of seeing the disease, I had made up my mind, from other circumstances than those furnished by reading and analogy, that it was so highly malignant in its nature, as to require an efficient course of stimulation from its commencement to its termination.

While attending lectures here five years ago, one of my roommates, Dr. R. G. Huntingdon, wrote his inaugural thesis upon this disease, very strenuously advocating the stimulating plan of treatment; having found it, he said, the most successful mode of cure in the complaint, as it had appeared in the eastern part of the Western Reserve in 1827.

I know, also, that my friend, Dr. Kirtland, of Trumbull Co., treated this disease, and the cynanche maligna, in the same manner;—and it was said, with better success, than those around him who stimulated. And I had not forgotten the fate of a relation, who was bled in this disease, by that Herculian practitioner of Hartford, Connecticut, Dr. Lemuel Hopkins.

By these means I was biased in favor of the stimulating plan of treatment in scarlatina—and it was not until I witnessed its various grades of action, that I became convinced that much of its malignity and mortality, was not inevitable, if *early* and judicious depletion were used. I found that its pathology was very similar to that of the malignant bilious fever of 1828—which was styled 'typhus syncopalis'—and rendered very mortal from being treated with stimulants. I concluded, therefore, to watch closely the several stages, and the progress of the excitement, so as to be prepared to vary the remedies as circumstances might require.

The difference of opinion among the physicians, with regard to the most proper mode of treatment, induced me to look at the result, which soon settled the question; the mortality under the antiphlogistic treatment being only one eighth as great, as that under the expectant or the stimulating treatment. This may appear like a broad assertion, but it can, nevertheless, be proved. As far as I could learn, some physicians did not bleed a single patient during the whole epidemic, except one or two at the very commencement of it.

The simple form of the disease required little treatment, except a cathartic or two, and good nursing. The inflammatory variety was the most frequent form during the summer and autumnal months, and required an efficient, well timed treatment, at the very outset of the excitement. The usual remedies were used for its stage of oppression—and when excitement developed itself, cathartics of calomel, and affusions of cold water, or cold water and vinegar, were applied to the surface.

When these failed to reduce the excitement sufficiently—and there was much tendency to local inflammation—venesection was resorted to. Where the eruption was profuse and general, it rarely became necessary to bleed—as a free eruption and internal inflammation did not seem capable of existing at the same time.

As soon as it becomes necessary to take notice of the affection of the throat—it is all-important that we apply externally some irritant of sufficient power to arrest the inflammation at once. A blister, mustard plaster, or spts. of ammonia, may be used, according to the severity of the local affection. It is very important that we attend to the affection of the tonsils early,—as it will then be an easy matter to subdue the affection—and prevent many dreadful consequences. I should mention, however, that an emetic of ipecacuanha, given early in the disease, when too much oppression is not present, will greatly relieve the inflammation of the throat—as well as most of the general symptoms—and it sometimes becomes necessary during the progress of the disease.

The best cathartic in this malady, especially during the summer and autumnal months, is calomel—and it should be used freely. At these times of the year the hepatic secretion is always found more or less disordered, and the bowels are generally torpid, and coated with a tenacious slime—so that the free use of calomel cannot well be dispensed with. It should be given in doses of from 8 to 20 grains—and permitted to remain in the system for several hours before any thing more is given to work it off. A few portions given in this manner, will evacuate the bowels, and reinstate the secretions, without effecting the mouth. The stools should always be inspected—and the calomel continued until the secretion of bile is found to be restored.

When the excitement is considerable, and the eruption free and general—cold effusions must not be neglected. Their effect is very beneficial, and they produce the most agreeable sensations to the patient. But when there is chilliness or local inflammation present, they must be withheld.

If the excitement run so high as to endanger the vitality of any important organ, from the supervention of inflammation or congestion, the lancet must be brought into requisition immediately--and it must not be laid aside, until the outward symptoms are entirely reduced—if we regard the subsequent well-being of our patient. We should bear in mind, at the same time, the diathesis of the system, and not unnecessarily waste the strength of the patient, as fatal determinations and prostration may occur at a moment when we little expect them.

It appears that the older authors advocated depletion in this disease, even in the most malignant forms,—and I can hardly account for the fact, that most of the faculty, or at least most of the later writers, advocate a contrary treatment.

We should not depend upon one or two favorite means. It is from the combined influence of several remedies, with the antiphlogistic regimen—that we are to expect the best success when we wish to diminish the general and local excesses of the arterial system, and ward off inflammation and subsequent debility.

It is one of the nicest points in the practice of physic, not to be too profuse or too sparing in our applications; but to make their united force exactly equivalent to the reduction of the prominent symptoms.

It was not unusual to find our patients, at the first visit, inclining to stupor, or in a state of delirium. I recollect being called to one, who had been laboring under delirium for fourteen hours. A slight and partial eruption was present, the surface was hot and dry, pulse quick, tense, and small—and a disposition to stupor prevailed. I did not think the boy would live, and requested that some other physician should be called in to my aid, but the parents being unwilling to do so, I concluded, after stating my doubts, and the course I should pursue, to commence the treatment of the case. This case occurred in the month of September, near the commencement of the severest part of the epidemic, and as there was so much difference of opinion with regard to the proper treatment, I feared, if I should not succeed, that an injury to myself and to the depleting plan of treatment would be the consequence. I commenced, however, with the belief, that no man should practice physic, who is not at all times fully prepared to risk his professional reputation, on the chance of saving his patient's life. I was under the necessity of bleeding him three times, and took 40 ounces, in all. This with the use of calomel purges, restored him in a very short time.

The treatment in the congestive variety was the same as directed for the congestive forms of other diseases—except blood-letting. I am inclined to think Dr. Armstrong too partial to this remedy, in congestive diseases.

In the malignant form, when depletion cannot be carried any further, the use of capsicum should be the more relied upon, than any other one remedy. In this disease, its virtues are too little appreciated. It should be given in the manner directed by Dr. Eberle in his Practice of Physic. When there is a tendency in the tonsils to gangrene, I have always found it of inestimable value.

In making the foregoing, very imperfect observations—my whole object has been to direct the attention of the faculty to the diathesis in this disease—its several grades of action—the necessity of closely observing its progress—and the great importance of early, efficient, and well-timed depletion in the inflammatory—and of counteraction and stimulation in the congestive variety.

*Cincinnati, March,* 1833.

